# Cancer risk susceptibility loci in a Swedish population

**DOI:** 10.18632/oncotarget.22687

**Published:** 2017-11-25

**Authors:** Wen Liu, Xiang Jiao, Jessada Thutkawkorapin, Hovsep Mahdessian, Annika Lindblom

**Affiliations:** ^1^ Department of Molecular Medicine and Surgery, Karolinska Institutet, Stockholm, Sweden

**Keywords:** genome-wide association study, haplotype association analysis, cancer syndrome, cancer risk, cancer predisposition

## Abstract

A germline mutation in cancer predisposing genes is known to increase the risk of more than one tumor type. In order to find loci associated with many types of cancer, a genome-wide association study (GWAS) was conducted, and 3,555 Swedish cancer cases and 15,581 controls were analyzed for 226,883 SNPs. The study used haplotype analysis instead of single SNP analysis in order to find putative founder effects. Haplotype association studies identified seven risk loci associated with cancer risk, on chromosomes 1, 7, 11, 14, 16, 17 and 21. Four of the haplotypes, on chromosomes 7, 14, 16 and 17, were confirmed in Swedish familial cancer cases. It was possible to perform exome sequencing in one patient for each of those four loci. No clear disease-causing exonic mutation was found in any of the four loci. Some of the candidate loci hold several cancer genes, suggesting that the risk associated with one locus could involve more than one gene associated with cancer risk. In summary, this study identified seven novel candidate loci associated with cancer risk. It was also suggested that cancer risk at one locus could depend on multiple contributing risk mutations/genes.

## INTRODUCTION

Although environmental factors explain most of cancer cases, inherited risk factors contribute to a various degree in development of different cancers [1]. Cancer syndromes are rare and typically involve families with early onset of disease. Most known cancer syndromes were first found to be associated with an increased risk of one tumor type such as breast cancer (*BRCA1* and *BRCA2*) or colorectal cancer (*APC* and the DNA mismatch repair genes) [2–6]. The *BRCA1* and *BRCA2* genes were first reported as breast cancer predisposing genes [2, 3]. However, it was soon clear that ovarian cancer and pancreatic cancer were also associated with the syndromes [7, 8]. Lynch Syndrome was first defined as a syndrome of hereditary non-polyposis colorectal cancer [5, 6]. It is today known to involve a broad spectrum of tumors [9]. In general, cancer syndromes confer an increased risk of not just one tumor type but rather constitute an inherited predisposition to many different types of cancer.

Recently, different approaches have been used to find new cancer syndromes. The Utah Population Database, including the record of cancer data for 190,000 individuals diagnosed with cancer in Utah from 1958, proved that many cancer sites showed a heritable contribution, which was associated with other cancer sites [10]. The Swedish Family-Cancer Database (FCD), first created in the 1990s, contains more than one million cancers. Studies using this database, reported that lung cancer had a significant association with 13 other cancer types and most of them were smoking related, such as bladder-, esophagus-, liver-, cervical- and kidney cancer [11]. Using the network of case and control studies from Italy and Switzerland, it was reported that several potential cancer syndromes appear among close relatives in an early age [12]. Based on the Icelandic Cancer Society (ICR) database, it was found that genetic factors are involved in several cancers and also play an important role in the familial clustering of cancer [13]. Stomach and prostate cancers were involved in most pairs of the tested cancer sites [13]. This is consistent with our finding in a recent study of colorectal cancer families [14].

The global analysis of genome-wide association studies (GWAS) have primarily focused on single diseases [15–17]. However, combining large-scale GWA meta-analysis, new loci have been found to be associated with an increased risk of hormone-related cancers such as breast-, ovarian- and prostate cancer [18]. We have already shown that familial colorectal cancer is associated with higher risk for other cancers and thus these families seem to segregate genetic risk factors for many different cancers [14]. To define a new cancer risk alleles predisposing to a variety of cancer types, we conducted a GWAS in 3,555 cancer cases and 15,581 controls. Sweden has a fairly homogenous population and Swedish founder mutations are known in disease genes. Thus, we hypothesized that novel loci with a founder mutation could be possible to detect using a Swedish GWAS. The definition for a founder mutation is that it occurs on the same specific haplotype in a population and thus we used haplotype analysis rather than single SNP analysis for more power to detect a founder effect.

## RESULTS

**Figure 1:**
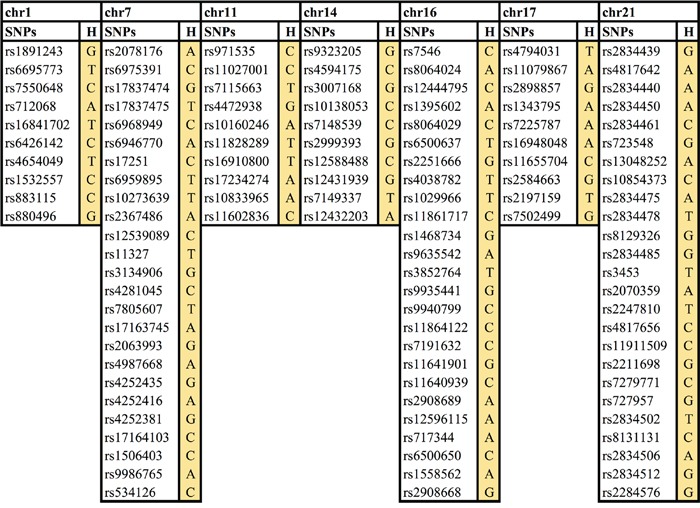
Seven novel cancer risk loci/haplotypes Chr, chromosome, H, haplotype.

Haplotypes describe the linear relationship of a series of loci along the chromosome strand and in PLINK defined by a certain number of single SNP markers. Two haplotype analyses on sliding windows of 10 and 25 SNPs using 3,555 cancer cases and 15,581 healthy controls were conducted. The statistical analysis suggested seven loci associated with cancer risk and with a p-value <1.1×10^−7^ (Figure 1). Four risk haplotypes were found using window 10, on chromosomes 1, 11, 14, and 17 while three risk haplotypes were found using window 25, on chromosomes 7, 16 and 21 (Figure 1).

Further analysis was performed around each of these seven haplotypes to find out which exact haplotype at each locus had the best p-value. For the loci on chromosomes 7, 11, 16 and 17 the first identified haplotypes were the most statistically significant, while for the loci on chromosomes 1, 14 and 21, a slightly shorter or longer haplotype were even more statistically significant (Table 1).

**Table 1:** Haplotype frequency and odds ratio for the seven loci

Chr	WS	HFA	HFC	OR	P Value	BWS	HFA	HFC	OR	P Value
1	10	0.012	0.007	1.67	8.49E-07	7	0.014	0.008	1.68	6.29E-07
7	25	0.010	0.005	1.9	3.03E-07	25	0.01	0.005	1.90	3.03E-07
11	10	0.021	0.013	1.61	6.13E-07	10	0.021	0.013	1.61	6.13E-07
14	10	0.029	0.020	1.45	4.52E-07	8	0.021	0.013	1.56	2.25E-07
16	25	0.011	0.006	1.83	3.01E-07	25	0.011	0.006	1.83	3.01E-07
17	10	0.041	0.030	1.35	7.27E-07	10	0.041	0.030	1.35	7.27E-07
21	25	0.013	0.030	0.41	4.73E-07	31	0.013	0.031	0.41	3.04E-07

Chr, chromosome; HFA, haplotype frequency in affected; HFC, haplotype frequency in controls; OR, odds ratio; WS, window size; BWS, best window size.

**Figure 2:**
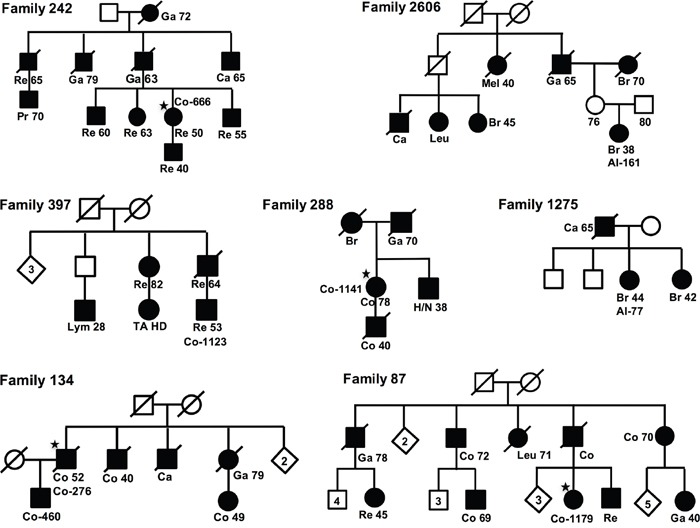
Pedigrees Pedigrees for the seven families, fulfilling one (of four) risk-haplotypes. For each family the case with the risk haplotype is indicated with sample-ID (★), and for all cases are shown diagnoses, Re, Rectal Cancer; Ga, Gastric Cancer; Br, Breast Cancer; Ca, Cancer; Co, Colon Cancer; Leu, Leukemia; Lym, Lymphoma; H/N, Head and Neck Cancer; Mel, Melanoma; Pr, Prostate Cancer.

All risk haplotypes had odds ratio (ORs) of 1.3-1.9, except the locus on chromosome 21, which had an OR of 0.4, thus the minor allele was associated with a decreased cancer risk. The 6 risk haplotypes were searched for among 104 familial cancer patients (from 58 breast cancer families and 46 colorectal cancer (CRC) families). Seven families had family members with the haplotypes on chromosomes 7, 14, 16 and 17 (Figure 2). Family 242 (Co-666) and family 397 (Co-1123) had the suggested haplotype on chromosome 7, family 87 (Co-1179) and family 1275 (Al-77) the haplotype on chromosome 14, family 134 (Co-276) the haplotype on chromosome 16, and family 288 (Co-1141) and family 2606 (Al-161) the haplotype on chromosome 17. Several other family members could have the haplotypes but were not fully informative for all markers (Supplementary Table 1). Some of the patients had more than one complete or incomplete haplotype.

Exome sequencing data were available for one patient with a full haplotype for the loci on chromosome 7, 14, 16 and 17 (Co-666 from family 242, Co-1179 from family 87, Co-460 from family 134 and Co-1141 from family 288). All non-exonic and synonymous variants, and those with a Minor Allele Frequency (MAF) >20% in 1000Genomes (1000G) or ExAC Non-Finnish European (NFE) were excluded.

For the locus on chromosome 7, three variants in the *PRSS1* gene, were found in the sample with the complete haplotype (Co-666). The first one, rs145867820 was also found in one other sample (Co-1053) with incomplete haplotype for this region. It was considered pathogenic by seven of nine bioinformatics functional tools. The frequency of the minor SNP allele (T) in ExAC and 1000G was 0.007 and 0.03, respectively, and in our 294 unrelated familial cases 0.05. This SNP was tested using TaqMan assay in 378 unrelated familial cancer cases and 379 controls and the result did not suggest any risk associated to this SNP (OR<1, frequency in cases 2% and in controls 3%). The second variant, rs200070487, was predicted to be pathogenic by all nine bioinformatics functional tools, the frequency of G allele was 0.01 in both ExAC and 1000G. None of the three patients (Co-1053, Co-851 and Co-700) with incomplete haplotypes had it. The frequency of the G allele among 294 unrelated familial cases was 0.20. It was not possible to generate a probe for this SNP for TaqMan assay. The third variant, rs200902389, was predicted pathogenic by one of the nine bioinformatics functional tools. Two patients from three other families with incomplete haplotypes also bore this variant (Co-700 and Co-1053). The frequency of the A allele in ExAC was 0.003 and in 1000G <0.01. The frequency of the A allele among 294 familial cases was 0.12. The TaqMan assay for this SNP failed. Thus, it could not be ruled out that rs200070487, or even rs200902389, could be associated with an increased risk.

The patient (Co-1179) with the chromosome 14 haplotype had no exonic variant. The patient (Co-460) with the chromosome 16 haplotype, had one variant in the *PPL* gene, which was considered too common (MAF=0.53) since the frequency of the risk haplotype was estimated to 1%. One patient with the chromosome 17 haplotype (Co-1141) had one variant in the *ZNF652* gene, the frequency of which in ExAC was 0.00002, and thus it was considered too rare to explain a risk haplotype with a frequency of 4%.

Next, analyses were undertaken to find out what haplotypes and possible loci were missed by our approach using only window sizes 10 and 25. A sliding window analysis consists of different sets of contiguous loci at various sliding positions [19]. The whole genome was searched with a sliding window strategy testing windows ranging from 1 to 25 markers. In total, nineteen additional loci showed a p-value less than 10^-6^ in different window sizes, and one haplotype on chromosome 13 even reached 6.45×10^-8^(window size 21, rs912593-rs9599474, OR=2) (Supplementary Table 2). None of these loci were considered statistically significant due to multiple testing.

Using the sliding window information within the seven suggested loci, including all windows with OR >1 and p <0.05, the analysis suggested that four of the risk loci could be attributed by more than one risk variant within the haplotype, such as for the loci on chromosomes 7, 16, 17 and 21, while the haplotypes on chromosomes 1, 11, and 14 suggested only one risk variant (Supplementary Figures 1-4).

## DISCUSSION

Our genome-wide haplotype association study using Swedish Twin Registry for 3,555 cancer cases and 15,581 healthy controls suggested seven loci associated with cancer risk (Table 1). All were rare (allele frequency 1-2%), and minor alleles of six of them were associated with increased cancer risks, while one was associated with a lower risk of cancer.

Comparing the six cancer risk haplotypes with our familial breast and colorectal cancer patients, seven families showed well-matched haplotypes for four of those risk loci, on chromosomes 7, 14, 16 and 17 (Figure 2 and Supplementary Table 1). By studying exome sequencing data from family members, we searched genes for those four haplotype regions. Although no clear disease-causing mutation was found, the data still supported the loci as risk susceptibility regions since at least four of the haplotypes were present in known cancer families. The causal variants may be located within regulatory regions. In fact, most risk loci identified from earlier GWASes have risk-associated SNPs far from genes, which were still considered relevant and potentially targeting adjacent genes or genetic elements such as RNAs or enhancer regions [15–18].

There are many genes located in the candidate regions on chromosome 7, such as *PRSS1, EPHB6, TRPV6, TRPV5 and PIP*. The genes *PRSS1*, *TRPV5* and *PIP* have so far been implicated in a few cancer types (pancreatic-, non-small cell lung- and breast cancer), while *EphB6* and *TRPV6* have been studied in relation to numerous cancer types. In this study, we found three missense variants in the *PRSS1* gene. One did not show any significant difference in allele frequency between cases and controls in subsequent validation, whereas the other two could not be ruled out, and need to be tested in further studies for conclusive results. *EphB6* overexpression together with *APC* gene mutations was suggested to promote the development of colorectal tumors [20]. A germline missense mutation in *EphB6* has been suggested to possibly predispose to familial CRC [21]. The same gene has been suggested to harbor driver mutations in melanoma [22], and the *Eph* family receptors have been implicated in tumor progression and clinical outcome in several malignancies including tongue squamous cell-, ovarian-, gastric-, breast-, non-small cell lung cancer, melanoma and neuroblastoma [23–29]. The *EphB6* has also been studied in thyroid-, and prostate cancer [30, 31]. Up-regulation of the TRPV6 Ca^2+^ channel in prostate cancer cells was suggested to promote cell proliferation rate, and to increase survival and apoptosis resistance in prostate cancer cells [32]. *TRPV6* was highly expressed in estrogen receptor-negative breast cancer cells, regulating their proliferation, suggesting that *TRPV6* can be a potential therapeutic target in these cancers [33]. Studies in candidate cancer genes including *EPHB6/TRPV6* found two SNPs in *EPHB6/TRPV6* marginally associated with survival in CRC [34]. The *TRPV6* gene has also been suggested to influence prognosis in cervical- and esophagus squamous cell cancer, as well as in non-small cell lung cancer [35–37]. Sliding window analysis suggested that more than one gene in this risk haplotype could contribute to the increased risk. (Supplementary Figure 1).

The *TMX1* gene, located in the risk region on chromosome 14, has been reported to be widely expressed in normal human tissues, and has been suggested to act as a tumor suppressor [38]. For this locus, the sliding window analysis suggested one contributing risk locus, involving the *TMX1* gene (Supplementary Figure 2).

The risk region on chromosome 16 contains candidate genes such as *PPL* and *GLYR1*. Primarily the *PPL* gene has been suggested to play a role in many cancers. The gene has an important role in skin tumor-protection [39]. *PPL* has also been reported to be relevant in prognosis in urothelial carcinoma of the urinary bladder-, colorectal -, esophageal-, endometrial cancer and triple-negative breast cancer [40–44]. Somatic mutations in the *GLYR1* have been suggested to drive tumorigenesis in microsatellite unstable tumors [45]. For this risk haplotype, sliding window analysis suggested at least two possible risk loci on the haplotype, involving the *PPL* and other genes (Supplementary Figure 3).

The region on chromosome 17 harbors two strong candidate cancer genes, *ZNF652, and PHB*. *ZNF652* is a known prostate cancer candidate gene. The protein expression in androgen receptor positive prostate cancer cells is associated with prostate cancer outcome and relapse [46]. It has also been suggested that *ZNF652* plays a role in the development of breast cancer [47] and vulvar squamous cell carcinoma [48]. *PHB* is evolutionarily conserved and plays an important role in human cellular senescence and tumor suppression [49]. A single nucleotide polymorphism has been suggested to increase the risk of breast cancer [50–52]. It has also been suggested to be implicated in prognosis in colorectal-, and bladder cancer [53, 54]. The *PHB* has also been suggested as a potential biomarker for gastric cancer and to be involved in prostate-, and papillary thyroid cancer [55–57]. For this candidate haplotype, the sliding window analysis suggested three, non-overlapping, possible risk loci contributing to the increased risk (Supplementary Figure 4).

The locus on chromosome 1 holds the gene *CNIH3*, not known to be involved in any type of cancer, and the locus on chromosome 11 harbors the non-coding RNA gene *RP11-266A24.1*. The chromosome 21 locus involves potentially interesting genes for cancer development/protection. *KCNE2* encodes a voltage-gated potassium channel ancillary subunit and is highly expressed in gastric parietal cells, and was suggested to suppress the proliferation of gastric cancer [58]. The *RCAN1* gene was up-regulated in cancer cells, resulting in inhibition of the cell motility, and *RCAN1* knockdown was suggested to promote thyroid cancer tumor growth [59].

This study design used only two window-sizes, 10 and 25, to explore possible novel cancer risk loci in a Swedish population. Analysis with window size ranging from 1 to 25 could demonstrate that another nineteen haplotypes could be of interest. Importantly, these analyses suggested that for some haplotypes (loci), the genetic risk could result from mutations related to more than one gene. Thus, it is possible that some of the candidate haplotypes hold mutations in two or even more of the candidate genes discussed above. If we had searched all windows up to 25 for the first study, only one would have been statistically significant because of multiple testing.

In conclusion, the strategy of haplotype analysis was facilitated by the fact that Sweden has a relatively homogenous population and identified seven novel candidate risk loci, with specific Swedish haplotypes to be associated with an increased risk of cancer. The result showed ORs higher than most previous GWASes, performed in mostly single cancer types, and using single SNP-analysis. However, these genetic risk loci should be relevant in all populations. It was also suggested that one haplotype could consist of more than one contributing cancer risk allele, possibly involving multiple genes. This could also be one reason why these loci have higher ORs compared to what is seen in many other, single-SNP related, GWASes. Further studies will be necessary to confirm these loci and risk association, and to find out what tumor spectrum is associated with these loci.

## MATERIALS AND METHODS

### Haplotype GWAS in 3,555 cancer cases and 15,581 healthy controls

The GWAS was based on two datasets (TwinGene and PsychArray) from the population-based Swedish Twin Registry [60]. Phenotypic data on cancer were obtained through linking the twins to the Swedish Cancer Registry using the unique person identification number available for all Swedish citizens. In this study, cases were cancer patients selected from twin pairs where at least one twin was affected by any type of cancer, and controls were selected from twin pairs where none was affected. Only one twin from each twin pair was included in the analysis.

### Familial cancer cases

Patients from families undergoing genetic counseling in the department of Clinical Genetics, Karolinska University Hospital have been recruited for genetic studies to find predisposing genes. Families with mutations in known cancer genes were excluded. Familial cancer cases were defined as coming from families where at least two first or second-degree relatives were affected with colorectal- or breast cancer. To be able to study haplotypes in familial cancer patients, a sample from at least on child or parent to each patient had been obtained.

### Samples used in Taqman experiment

TaqMan experiment test candidate variants used 378 familial cases described above and 379 controls from the Swedish Colorectal Cancer Low-Risk Study [14], which recruited consecutive colorectal cancer cases and their spouses as controls.

### Genotyping and quality control (QC) of twins

Genome DNA was extracted from peripheral blood samples for both the cases and the controls using standard procedures. In the TwinGene study, DNA from 9,836 individual subjects was genotyped with Illumina OmniExpress bead chip; whereas in PsychArray, 18,560 twins were genotyped using the Illumina Infinium PsychArray-24 BeadChip. Both studies include all available dizygotic twins and one twin in each pair of monozygotic twins.

For the quality control (QC) of the TwinGene study, variants were excluded from analysis if call rate was <=0.97, minor allele frequency was <1% or if the variant deviated significantly from Hardy-Weinberg equilibrium (p <=1×10^−7^). Samples were removed in case of genotyping success rate <97%, gender discrepancy between reported and X-chromosome heterozygosity-predicted, abnormal heterozygosity (>3 standard deviations from mean) or detection of cryptic relatedness. In total, 9617 individuals and 644556 SNPs remained after the QC. And in the PsychArray study, variants were excluded from analysis if call rate was <0.98, cross-batch discordance >10%, more than one discordant genotype within monozygotic twin pairs, deviated significantly from Hardy-Weinberg equilibrium (p <1×10^−7^), significantly associated with more than one genotyping batch (at p <5×10^−8^), the variant calling was poor (Y-chromosomal or mitochondrial) or the allele frequency differed by >10% (absolute difference) from that of 1000 Genomes European samples and mean GenCall scores are <0.5. Samples were excluded in case of genotyping success rate <98%, gender discrepancy between reported and X-chromosome heterozygosity-predicted, abnormal heterozygosity (autosomal inbreeding coefficient F outside ±0.2), possible sample contamination (relatedness with other samples >6 standard deviations from mean in a random set of 1000 samples) or evidence of non-European ancestry (>6 standard deviations from the mean values of the first two principal components in 1000 Genomes European population). 17,898 individuals and 561,187 markers passed the QC.

### Quality control of the merged data set

To be used in this association study on cancer risk, the TwinGene study (2,457 cancer cases and 4,441 controls) were merged with the PsychArray study (1,099 cancer cases and 11,239 controls) and analyzed for the 237,799 markers existing in both datasets using PLINK [61]. In the analysis, 59 markers were removed due to inconsistent strand coding. Therefore, a total of 19,236 individuals (3,556 cases and 15,680 controls) and 237,740 markers were included in the analysis. Next, heterozygous haploid genotypes were excluded as well as samples with gender inconsistency and same position variants. In the next stage, 237,740 SNPs and 19,236 individuals (3,556 cases and 15,680 controls) were merged and SNPs with <98% call rate, <5% minor allele frequency (MAF), and those inconsistent with Hardy–Weinberg equilibrium in controls, were removed. In the final stage, 226,883 SNPs and 19,236 individuals (3,556 cases and 15,680 controls) remained and a multidimensional scaling (MDS) analysis was conducted on all the remaining markers for the purpose of population stratification and to identifying ethnic outliers. These outliers were excluded from the dataset while the remaining were plotted in an MDS plot (Supplementary Figure 5). In the end, 226,883 SNPs and 19,136 individuals (3,555 cases and 15,581 controls) remained for further downstream analyses.

### Genotyping of familial samples for testing of haplotypes

Genomic DNA was extracted from peripheral blood using standard procedures. Genotyping of a total of 587 individuals, familial CRC cases and their relatives, was performed by the Illumina Infinium assay using the Illumina HumanOmniExpress-12v1_H BeadChip. The results on 730,525 SNPs, were analyzed using the software GenomeStudio 2011.1 from Illumina Inc. Average sample call rate per SNP with sample call rate >0 was >99% and the overall reproducibility >99.99%. Arrays were processed according to manufactures’ protocol at the SNP&SEQ Technology Platform at Uppsala University and is available on request (www.genotyping.se).

### Exome sequencing

Genome DNA extracted from peripheral blood was quantified using the Qubit Flurometer (Life Technologies). Sequencing libraries were prepared according to the TruSeq DNA Sample Preparation Kit EUC 15005180 or EUC 15026489 (Illumina). 1-1.5 μg of genomic DNA was fragmented using a Covaris sonicator (Covaris, Inc.). Thirty-seven of the DNA samples were fragmented according to the Covaris 400bp protocol and sixty-one samples were fragmented according to the SureSelect Protocol. After the fragmentation, all samples were subjected to end-repair, A-tailing, and adaptor ligation of Illumina Multiplexing PE adaptors. An additional gel-based size selection step was performed for the 37 samples. The adapter-ligated fragments were subsequently enriched by PCR followed by purification using Agencourt AMPure Beads (Beckman Coulter). Exome capture was performed by pre-pooling equimolar amounts and performing enrichment in 5- or 6-plex reactions according to the TruSeq Exome Enrichment Kit Protocol (EUC 15013230). The library size was checked on a Bioanalyzer High Sensitivity DNA chip (Agilent Technologies) while concentration was calculated by quantitative PCR. Pooled DNA libraries were clustered on a cBot instrument (Illumina) using the TruSeq PE Cluster Kit v3. Paired-end sequencing was performed for 100 cycles using a HiSeq 2000 instrument (Illumina) with TruSeq SBS Chemistry v3, according to the manufacturer's protocol. Base calling was performed with RTA (1.12.4.2 or 1.13.48) and the resulting BCL files were filtered, de-multiplexed, and converted to FASTQ format using CASAVA 1.7 or 1.8 (Illumina). Data were analyzed using the bcbb package (https://github.com/chapmanb/bcbb). After sequencing, the reads were aligned to the reference genome hg19 (GRCh37) using BWA, sorted and PCR duplicates were removed with Picard. The calculation of mapping and enrichment statistics were done with Picard and GATK. Variants were called using GATK and followed by a best practice procedure implemented at the Broad Institute [62].

### Mutation annotation

The output mutations in variant call format (vcf) were annotated using ANNOVAR [63], which generated an Excel-compatible file with gene annotation, amino acid change annotation, dbSNP identifiers [64], and 1000 Genomes Project allele frequencies [65].

### Functional prediction

Pathogenicity of variants was predicted by nine bioinformatics functional tools which were: SIFT, Polyphen2_HDIV (Polymorphism Phenotyping v2), Polyphen2_HVAR, LRT, Mutation Taster, Mutation Assessor, FATHMM (Functional Analysis through Hidden Markov Models), RadialSVM, LR (Likelihood Ratio test).

### TaqMan assay

A total of 378 cancer cases and 379 controls were genotyped using TaqMan genotyping Assay (Applied Biosystems, Foster City, CA, USA). Primer of rs145867820's context sequence[VIC/FAM]:CACCATGCCTGCCCTGCCCATCAGC[C/T]GCATCCAGGTGAGA CTGGGAGAGCA. Primer of rs200902389's context sequence [VIC/FAM]: CCCTGTGGTCTGCAATGGACA GCTCCAAGGA[G/A]TTGTCTCCTGGGGTGATGGCT GTGCCCAGA.

### Statistical analysis

A logistic regression model was employed to examine the association between one single SNP or a haplotype and cancer risk. Corresponding OR, standard errors, 95% confidence intervals and P values were calculated accordingly using PLINK v1.07 [61]. An MDS plot showing P values sorted by chromosomal position was generated to provide a visual illustration of top association findings across the genome. Bonferroni-adjusted P value criteria for genome-wide statistical significance of SNP was p < 1.1×10^−7^.

## SUPPLEMENTARY MATERIALS FIGURES AND TABLES


