# Supplementary material for: Cancer risk susceptibility loci in a Swedish population
Source: Oncotarget. 2017 Nov 25;8(66):110300–10. doi: 10.18632/oncotarget.22687 (PMC5746383; doi:10.18632/oncotarget.22687)
Supplement: Supplementary file 1 [file oncotarget-08-110300-s001.pdf]

Cancer risk susceptibility loci in a Swedish population

SUPPLEMENTARY MATERIALS

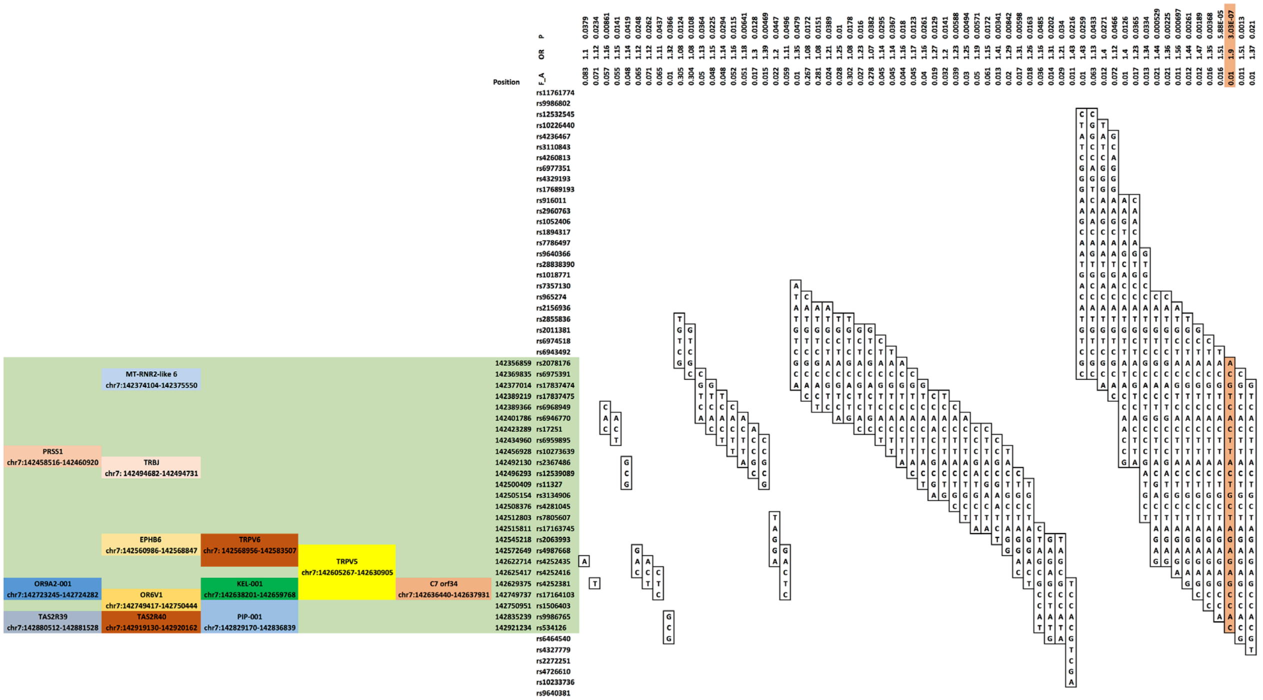

Supplementary Figure 1: Sliding window analysis for the locus on chromosome 7. All haplotypes from sliding window analysis with OR >1 and p-value <0.05. The statistics significant haplotype indicated in brown. The genes involved in the locus for the haplotype are shown. F_A, frequency in patients; OR, odds ratio.

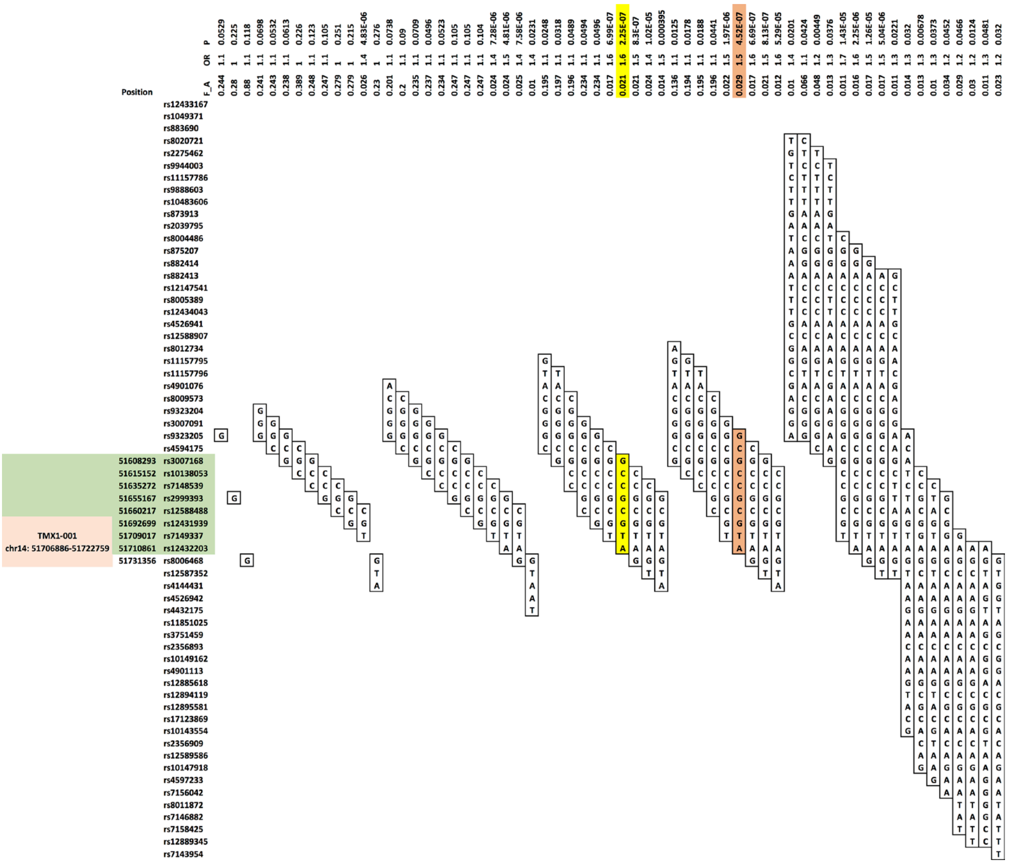

Supplementary Figure 2: Sliding window analysis for the locus on chromosome 14. All haplotypes from sliding window analysis with OR >1 and p-value <0.05. The statistics significant haplotype indicated in brown. Sliding window analysis window 8 in yellow with best p-value. The gene involved in the locus for the haplotype are shown. F_A, frequency in patients; OR, odds ratio.

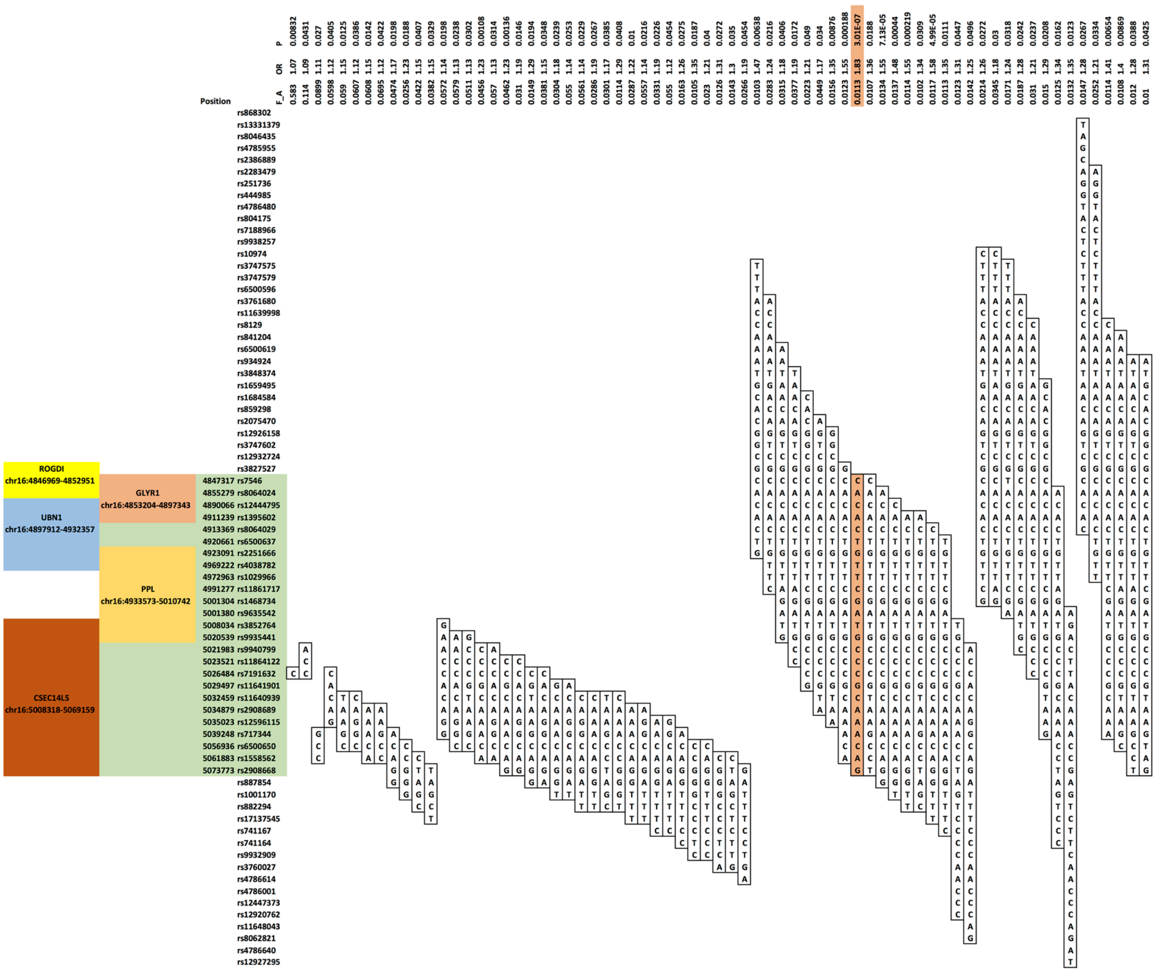

Supplementary Figure 3: Sliding window analysis for the locus on chromosome 16. All haplotypes from sliding window analysis with OR >1 and p-value <0.05. The statistics significant haplotype indicated in brown. The genes involved in the locus for the haplotype are shown. F_A, frequency in patients; OR, odds ratio.

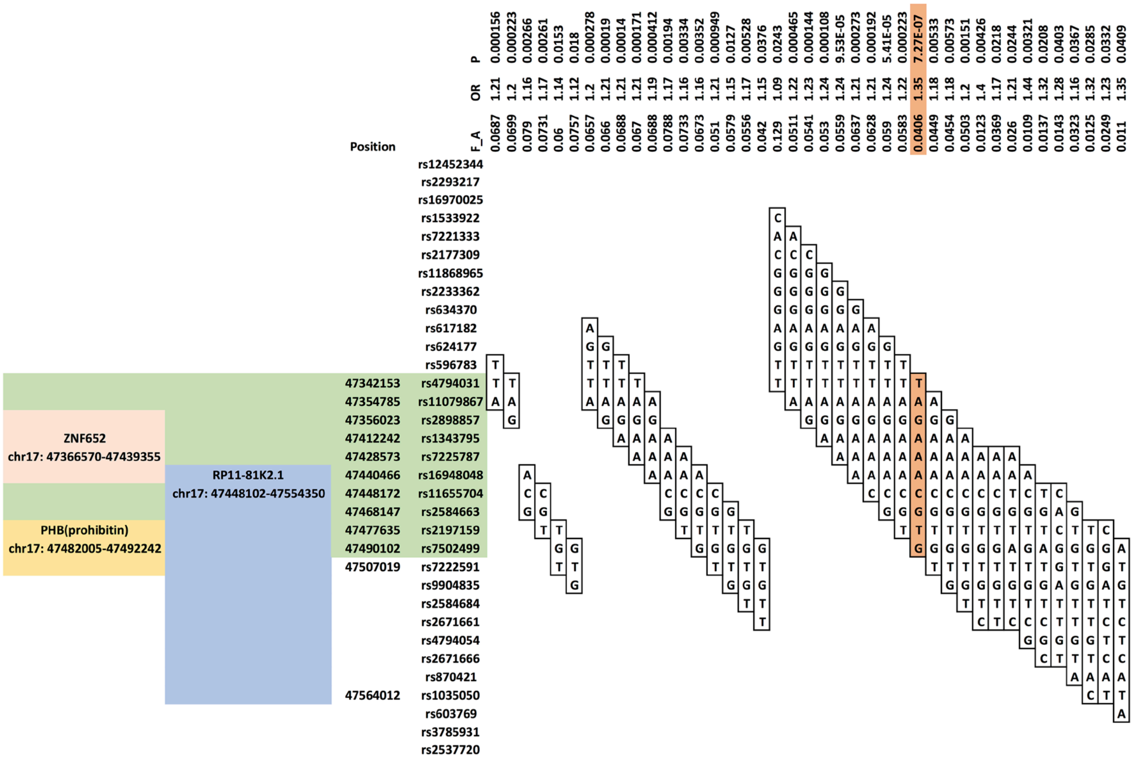

Supplementary Figure 4: Sliding window analysis for the locus on chromosome 17. All haplotypes from sliding window analysis with OR >1 and p-value <0.05. The statistics significant haplotype indicated in brown. The genes involved in the locus for the haplotype are shown. F_A, frequency in patients; OR, odds ratio.

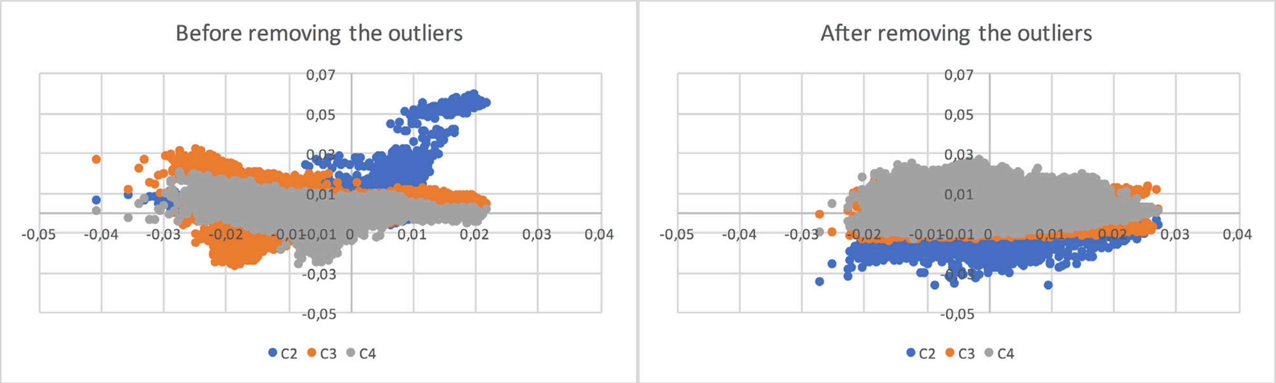

Supplementary Figure 5: Multidimensional scaling (MDS) analysis. Multidimensional scaling (MDS) analysis was conducted on all the markers for the purpose of population stratification and to identifying ethnic outliers. These outliers were excluded from the dataset while the remaining were plotted in an MDS plot. The scaling is based on covariate 1 (C1) and covariates C2, C3 and C4 which represented the position on first, second, third and fourth dimension respectively. Plotting the C1 values against C2, will give a scatter plot in which each point is an individual, and the two axes correspond to a reduced representation of the data in two dimensions, which can be useful for identifying any clustering.

Supplementary Table 1: Familial cancer patients as potential carriers of the six risk haplotypes

		chr1	chr7	chr11	chr14	chr16	chr17
**Famliy members with fully matched haplotypes**	**CRC**		397: **Co-1123**		87: **Co-1179**	134: **Co-276**	288: **Co-1141**
			242: **Co-666**				
	**Br**				1275: **Al-77**		2606: **Al-161**
**Famliy members with suggested but Incompleted haplotypes**	**CRC**	409: **Co-1254**	91: **Co-700**	**155: Co-557**	12: **Co-90**	103: **Co-257**	12: **Co-90**
			301: **Co-1053**	**208: Co-1094**	103: **Co-257**	309: **Co-783**	68: **Co-201**
			325: **Co-851**	**288: Co-1141**	155: **Co-557**	397: **Co-1123**	309: **Co-783**
				**409: Co-1254**	216: **Co-367**	547: **Co-1621**	340: **Co-831**
					227: **294-89D, Co-365**		660: **Co-1599**
					237: **Co-452**		
					547: **Co-1621**		
					1085: **Co-1518**		
	**Br**	179: **Co-323**	1227: **Al-169**	**1898: Al-92**	1505: **Al-111**	1275: **Al-77**	79: **Al-65**
		4001: **Br-48**		**5020: Br-114**	3006: **And23**	2606: **Al-161**	1227: **Al-169**
		4038: **Br-53**		**6002: Co-328**	3009: **ING31**	6050: **Br-331**	1898: **Al-92**
		6002: **Co-328**		**6039: Br-312**	4038: **Br-53**	6076: **Br-399**	2101: **Al-176**
		6039: **Br-312**			5042: **Br-183**		6106: **Br-484**
		6080: **Br-409**			6022: **Br-289**		
					6082: **Br-419**		
					6089: **Br-432**		
					6103: **Br-499**		

Family members tested for six risk haplotypes, seven patients with fully matched haplotypes and 44 patients with incomplete matching haplotypes. For each patient is given family ID and patients ID, patients ID's in bold. Br, breast cancer families. CRC, colorectal cancer families.

Supplementary Table 2: Additional 19 possible risk haplotypes

Chr	WS	SNPs	Position (GRCh37)	OR	P Value
2	11	rs6756590-rs11686241	217208571-217298205	0.604	8.37E-07
3	17	rs10510776-rs7613572	55196913-55290746	1.75	1.35E-07
3	21	rs884828-rs9851042	168234218-168583606	0.395	6.10E-07
4	23	rs3018066-rs450533	107289526-107830261	1.62	7.17E-07
4	14	rs7684187-rs6844999	123341159-123581235	1.7	6.48E-07
5	17	rs4869272-rs1498924	95539448-95779230	0.831	2.46E-07
5	22	rs4835937-rs469722	127337086-127666565	1.86	4.92E-07
5	19	rs10035650-rs153852	93936485-94176581	1.54	5.50E-07
6	23	rs9377188-rs659119	149351381-149533147	1.39	5.10E-07
6	15	rs5029614-rs3476	18100374-18225173	1.8	6.85E-07
6	7	rs719150-rs9376307	138192761-138283265	0.737	1.73E-07
8	21	rs2953950-rs2912500	69693543-69944403	1.34	7.39E-07
9	21	rs7856656-rs2182318	37421065-37641833	1.39	9.91E-07
9	24	rs7863342-rs3763608	71473803-71647375	1.56	5.36E-07
10	14	rs1577074-rs10490913	119983056-120154436	1.81	8.90E-07
11	7	rs538592-rs4121859	95101147-95178140	1.57	1.78E-07
12	22	rs4553443-rs12580194	55323545-55696558	1.28	7.17E-07
13	21	rs912593-rs9599474	35032491-35295455	2	6.45E-08
18	24	rs4800995-rs2902193	53415377-53650309	1.53	6.89E-07

Chr, chromosome; WS, window size; OR, odds ratio.
